# Constructing Tumor Progression Pathways and Biomarker Discovery with Fuzzy Kernel *Kmeans* and DNA Methylation Data

**Published:** 2008-01-25

**Authors:** Zhenqiu Liu, Zhongmin Guo, Ming Tan

**Affiliations:** 1 Division of Biostatistics, University of Maryland Greenebaum Cancer Center, 22 South Greene Street, Baltimore, MD 21201, U.S.A; 2 Molecular and Structural Biology, University of Maryland Greenebaum Cancer Center, 655 W. Baltimore St., Baltimore, MD 21201. U.S.A

**Keywords:** progression pathway, kernel kmeans, fuzzy kernel kmeans, biomarkers

## Abstract

Constructing pathways of tumor progression and discovering the biomarkers associated with cancer is critical for understanding the molecular basis of the disease and for the establishment of novel chemotherapeutic approaches and in turn improving the clinical efficiency of the drugs. It has recently received a lot of attention from bioinformatics researchers. However, relatively few methods are available for constructing pathways. This article develops a novel entropy kernel based kernel clustering and fuzzy kernel clustering algorithms to construct the tumor progression pathways using CpG island methylation data. The methylation data which come from tumor tissues diagnosed at different stages can be used to distinguish epigenotype and phenotypes the describe the molecular events of different phases. Using kernel and fuzzy kernel *kmeans*, we built tumor progression trees to describe the pathways of tumor progression and find the possible biomarkers associated with cancer. Our results indicate that the proposed algorithms together with methylation profiles can predict the tumor progression stages and discover the biomarkers efficiently. Software is available upon request.

## 1. Introduction

DNA methylation is a post-replication modification predominantly found in cytosines of the dinucleotide CpG that is infrarepresented throughout the genome except at small regions named CpG islands. It is noted that methylation events responsible for silencing critical tumor suppressor genes that lead to tumorigenesis can be captured in the DNA epigenetic code of a tumor. As DNA is heritable and stable, retrospective methylation analysis on samples collected earlier tends to provide more complete clinicopathological information. A few studies (Baylin, 2005; Robertson, 2005; Yan et al. 2001, Chen et al. 2003, Wei et al. 2002) have reported that normally unmethylated CpG island (a short stretch of DNA in which the frequency of the CG sequence is higher than other region) located in the promoter region of cancer cells undergoes dense hypermethylation during tumor progression. Hypomethylation can also be found at different parts of the genome in cancer. The extent of both DNA hypomethylation and hypermethylation in the tumor cell is likely to reflect distinctive biological and clinical features. Researchers found that DNA hypermethylation and hypomethylation are independent processes and appear to play different roles in tumor progression (Frigola et al. 2005). Methylation of the CpG sites influences the activity of nearby genes and is critical to the regulation of gene expression. With the advances in high throughput technology, we can get the methylation signature of multiple genes simultaneously and classify tumors based on the global patterns of DNA methylation. In this research, we intend to show that solid tumor progression can be characterized by the progressive accumulation of epigenetic events.

We will concentrate on the hypermethylation analysis in this paper, even the proposed method can be applied to hypomethylation with little change. Several challenges we have to face in collecting and analyzing the multilocus hypermethylation data. First tumor tissues from different patients at different progression stages with distinct phenotypes have to be studied since it is difficult to collect tumor tissues of the same patient at different stages. Second we need to define an appropriate similarity (distance) measure before we can cluster the epigenetic data and construct the pathway. Wang et al. (2006) have used the weighted Euclidian distance to define the similarity of the binary hypermethylation data, which is not quite appropriate. Finally we have to build the tumor progression pathway with both methylation and phenotype data and each node (cluster) should make biological sense.

Directed acyclic graphs (DAGs) or tree diagrams (Wang et al. 2007) have been used to represent possible tumor progression pathway. The main objectives of pathway construction is to construct patterns and relationships among hypermethylated genes that are progressively accumulated during tumorigenesis. Therefore the phenotypes of the progeny node in the pathway tree are supposed to be more aggressive than the parent nodes and the parent’s hypermethylated loci are inherited by their progeny nodes.

In this paper, we proposed a two-step pathway construction algorithm. First we define a similarity measure using normalized mutual information and cluster the data with the measure to find the pathway nodes. Second we build the pathway tree based on the center of each cluster and the heritability of the genotype and phenotype data. By first clustering the genotype data, one reduces the number of multiple comparisons substantially. Moreover it is much more robust and more likely to be platform independent to look at aggregates of genes (clusters). Most importantly combining the genotypes along pathways is biologically meaningful. Pathways are closer to the clinical phenotype than the individual constituents of these pathways. The whole is usually more than the sum of its parts.

This paper is organized as follows. In section 2, we develop the clustering procedures for pathway construction. We construct a pathway and discover the associated biomarkers with real data in section 3. Conclusions and discussions are given in section 4.

## 2. Methods

### Kernel based clustering

Data clustering algorithms partition the data into pre-specified number of groups such that a well defined cost function is minimized. Many clustering algorithms have been developed in recent years. The most well-known and widely used algorithm is the iterative relocation scheme of Euclidean *kmeans* (Jain and Dubes, 1988). The popularity of this algorithm stems from its simplicity and scalability. However the squared-Euclidean cost function of *kmeans* is not a good match with the data and consequently *kmeans* performs poorly as compared with other approaches (Strehl, 2000). Another major drawback of *kmeans* is that it can not separate clusters that are nonlinearly separable in the input space. All of these have lead to the search for more appropriate distance (similarity) functions (Aggarwal, 2003; Strehl et al. 2000). Entropy based distance measure has been used for text classification (Dhillon et al. 2003).

Data produced by methylation array are binary in nature (1: hypermethylated; 0: unmethylated) for which clustering based on Euclidean distance is not meaningful. In this paper we propose the mutual information based distance (similarity) function which capture the nonlinear correlation between the variables. We define an entropy kernel by introducing the normalized mutual information. Mutual information also called Kullback-Leibler (KL) divergence (Kullback and Leibler, 1951) was first proposed to measure the distance between two probability distributions. Unlike correlation coefficient, mutual information captures both the linear and nonlinear correlations. Give two sequences **x** and **y**, the normalized mutual information is defined as (Liu and Chen, 2005):

(1) $$K(x,y)=\frac{\Sigma_{i,j}p(x_{i},y_{j})\text{log}\frac{p(x_{i},y_{j})}{p(x_{i})p(y_{j})}}{\text{min}\left\{h(x),h(y)\right\}},$$

where $h(x)=-\Sigma_{i}p(x_{i})\text{log}\frac{1}{p(x_{i})}$ is the entropy for sequence **x** and *h*(**y**) is the entropy for sequence **y** and 0 ≤ *K*(**x**,**y**) ≤ 1. *K*(**x**,**y**) = 0 when two sequences are independent and *K*(**x**,**y**) = 1 when they are completely correlated. *K*(**x**,**y**) is also symmetric with respect to the two sequences **x** and **y**.

It follows from the Mercer’s theorem that Equation (1) defines a kernel function. To form a kernel function, data points are mapped to a higher-dimensional feature space using a nonlinear function *φ*(**x**), then a kernel function is formed with the inner product, *K*(**x**,**y**) = *φ*(**x**)*^t^φ*(**y**), where Φ(**x**)*^t^* is the transpose of *φ*(**x**). However we can define a kernel function directly as in Equation (1) without knowing the transformation function *φ*(**x**) explicitly. Kernel *kmeans* partitions the data in the new feature space with the kernel matrix.

Given a set of input data **x**_1_, **x**_2_,..., **x***_n_*, the kernel *kmeans* algorithm seeks to find clusters C_1_,C_2_,...,C*_k_* that minimize the objective (error) function:

(2) $$E\left({\left\{C_{p}\right\}}_{p=1}^{k}\right)=\sum_{p=1}^{k}\sum_{{\text{x}}_{i}\in C_{p}}{\left\|\varphi ({\text{x}}_{i})-{\text{m}}_{p}\right\|}^{2},$$

where

$${\text{m}}_{p}=\frac{\Sigma_{{\text{x}}_{i}\in C_{p}}\varphi ({\text{x}}_{i})}{|C_{p}|}$$

Note that the *p*-th cluster is denoted by a clustering of the data by {*C_p_*}*_p =_* _1_*^k^*, and the mean of cluster *C_p_* by **m***_p_*. We obtain the distance computation with each center as follows:

(3) $$\begin{array}{l} d(x_{i},m_{p})={\left\|\varphi (x_{i})-m_{p}\right\|}^{2}=\varphi {\left(x_{i}\right)}^{t}\varphi (x_{i})\\ -\frac{2\Sigma_{{\text{x}}_{j}\in C_{p}}\varphi {\left(x_{i}\right)}^{t}\varphi (x_{j})}{|C_{p}|}\\ +\frac{\Sigma_{{\text{x}}_{j}\in C_{p}}\Sigma_{{\text{x}}_{l}\in C_{p}}\varphi {\left(x_{j}\right)}^{t}\varphi (x_{l})}{|C_{p}{|}^{2}}\\ =K_{ii}-\frac{2\Sigma_{{\text{x}}_{j}\in C_{p}}K_{ij}}{|C_{p}|}\\ +\frac{\Sigma_{{\text{x}}_{j}\in C_{p}}\Sigma_{{\text{x}}_{l}\in C_{p}}K_{jl}}{|C_{p}{|}^{2}}. \end{array}$$

Note the final result of Equation (3) is associated with kernel values only. It can be shown that kernel *kmeans* algorithm is equivalent to spectral clustering and graph partitioning (Dhillon et al. 2005). Spectral clustering is a eigenvector-based algorithm with kernel principal analysis, which can be computationally prohibitive. It also has the drawback that the number of eigenvectors used has to be predetermined. The equivalence of kernel *kmeans* and kernel principal component clustering implies that we can use the iteration algorithms for directly minimizing normalized-cut of graph. Therefore kernel *kmeans* is more computational efficient. With Equation (3), the standard *kmeans* iteration procedure can be applied without any difficulty. Details of the algorithm are given below:

### Kernel *Kmean* algorithm

- Calculating the kernel matrix *K* with the epigenetic data. Given the number of clusters *k*, the optional maximum number of iterations, and optional initial clusters:
  1. Initialize the k clusters *C*_1_^(0)^,…,*C_k_*^(0)^ randomly if no initial clustering is given. Set t = 0.
  2. for each sample x*_i_* and each cluster *p*, compute
    $$\begin{array}{l} d(x_{i},m_{p})=K_{ii}-\frac{2\Sigma_{{\text{x}}_{j}\in C_{p}}K_{ij}}{|C_{p}|}\\ +\frac{\Sigma_{{\text{x}}_{j}\in C_{p}}\Sigma_{{\text{x}}_{j}\in C_{p}}K_{jl}}{|C_{p}{|}^{2}}. \end{array}$$
  3. Update the clusters through sending **x***_i_* to cluster p* such that p* = arg min*_p_* *d*(**x***_i_*, **m***_p_*).
  4. continue *t* = *t* + 1 until **m***_p_* is converged or the maximum iteration is exceeded.
- Output the final clusters.

### Fuzzy kernel *kmeans*

The hard partitioning kernel *kmeans* is simple and popular, but its results are not always re liable and these algorithms have numerical problems as well. Fuzzy kernel *kmeans* algorithm is a generalization of kernel kmean. It minimizes the following error function:

$$E\left({\left\{C_{p}\right\}}_{p=1}^{k}\right)=\sum_{p=1}^{k}\sum_{i=1}^{n}{\left(\mu_{pi}\right)}^{q}{\left\|\Phi (x_{i})-m_{p}\right\|}^{2}$$

with constraints:

$$\sum_{p=1}^{k}\mu_{pi}=1,$$

where *μ_pi_* represents the membership degree and the weighting exponent *q* > 1 indicates the fuzziness of the clusters (*q* = 2 in our application). The function is minimized only if

(4) $$\mu_{pi}=\frac{1}{\Sigma_{j=1}^{k}{\left(d_{pi}/d_{ji}\right)}^{1/(q-1)}},\;\;\;1\leq p\leq k,\;\;\;1\leq i\leq n$$

and

(5) $$m_{p}=\frac{\Sigma_{i=1}^{n}\mu_{pi}^{q}\Phi ({\text{x}}_{i})}{\Sigma_{i=1}^{n}\mu_{pi}^{q}},\;\;\;1\leq p\leq k.$$

We have the squared distance

(6) $$\begin{array}{l} d_{pi}=d(x_{i},m_{p})={\left\|\varphi ({\text{x}}_{i})-m_{p}\right\|}^{2}=\varphi {\left(x_{i}\right)}^{t}\varphi (x_{i})\\ -\frac{2\Sigma_{j=1}^{n}\mu_{pj}^{q}\varphi {\left(x_{i}\right)}^{t}\varphi (x_{j})}{\Sigma_{j=1}^{n}\mu_{pj}^{q}}\\ +\frac{\Sigma_{j=1}^{n}\Sigma_{l=1}^{n}\mu_{pj}^{q}\mu_{pl}^{q}\varphi {\left(x_{j}\right)}^{t}\varphi (x_{l})}{{\left(\Sigma_{j=1}^{n}\mu_{pj}^{q}\right)}^{2}}\\ =K_{ii}-\frac{2\Sigma_{j=1}^{n}\mu_{pj}^{q}K_{ij}}{\Sigma_{j=1}^{n}\mu_{pj}^{q}}\\ +\frac{\Sigma_{j=1}^{n}\Sigma_{l=1}^{n}\mu_{pj}^{q}\mu_{pl}^{q}K_{jl}}{{\left(\Sigma_{j=1}^{n}\mu_{pj}^{q}\right)}^{2}}. \end{array}$$

### The algorithm

- Calculating the kernel matrix *K* with the epigenetic data. Given the number of clusters *k*, the optional maximum number of iterations, and the termination tolerance ɛ > 0.
  1. Initialize the partition matrix *U*^0^ = [*μ_pi_*]*_k^×n^_*, and set t = 0.
  2. Compute the distances:
    $$d_{pi}=K_{ii}-\frac{2\Sigma_{j=1}^{n}\mu_{pj}^{q}K_{ij}}{\Sigma_{j=1}^{n}\mu_{pj}^{q}}+\frac{\Sigma_{j=1}^{n}\Sigma_{l=1}^{n}\mu_{pj}^{q}\mu_{pl}^{q}K_{jl}}{{\left(\Sigma_{j=1}^{n}\mu_{pj}^{q}\right)}^{2}}.$$
  3. Update the partition matrix:
    $$\mu_{pi}^{t}=\frac{1}{\Sigma_{j=1}^{k}{\left(d_{pi}/d_{ji}\right)}^{1/(q-1)}}$$
- Stop until ||U*^t^* − U*^t^*^−1^|| < ɛ

### Cluster validation

The number of clusters is predefined in the two kernel *kmeans* algorithm. There is no generally accepted procedure for determining the number of clusters. This decision should be guided by theory and practicality of the results, along with the use of the inter-cluster distances at successive steps. The distance between two samples can be calculated with *d*(**x***_i_*, **x***_j_*) = *K*(**x***_i_*, **x***_i_*) + *K*(**x***_j_*, **x***_j_*) − 2*K*(**x***_i_*, **x***_j_*). One approach to determining the number of clusters is to utilize the validation function. Different validity measures have been proposed in the literature, non of them is perfect by itself. We have utilized Dunn’s index (DI) to determine the number of clusters.

$$DI(k)=\underset{i\in k}{\text{min}}\left\{\underset{j\in k,i\neq j}{\text{min}}\left\{\frac{{\text{min}}_{x\in C_{i},y\in C_{j}}d(x,y)}{{\text{max}}_{p\in k}\{{\text{max}}_{x,y\in C_{p}}d(x,y)\}}\right\}\right\}.$$

The number of clusters is chosen with the maximal DI value.

## 3. Pathway Discovery

The clusters found with kernel *kmenas* can be used as the nodes of the pathway tree. We can build the pathway with the centers of genotype and phenotype data of each cluster. Because both genotype and phenotype data are ordinal in nature, their centers are defined to be the medians of each cluster. Given K genotype centers {GC*_i_*}*_i_* _= 1_*^K^* and phenotype centers {PC*_i_*}*_i_* _=_*^k^*, the tumor progression tree are built with the heritability of each node, i.e. a child node *j* from its parent node *i* must satisfy G*C_j_* ≥ G*C_i_* and PC*_j_* ≥ P*C_i_*. Under this condition, all hypermethylated loci in a parent node is inherited by its progeny node and the progeny node has at least the same serious phenotype condition as its parent node.

### Computational experiments

Our experiments are performed with the methylation data of 50 breast carcinomas of unrelated patients (http://www.stat.ohio-state.edu/statgen/). There are 9 genes for their methylation status (0: unmethylated; 1: hypermethylated). The 9 genes are: GPC3 RASSF1A, WT1, uPA, HOXA5, p16, 3OST3B, BRCA1, and DAPK1. The 4 phenotype measurements used in our analysis are ER/PR (1: +/+; 2: +/− 3; −/−), histology (1: well-differentiated, WD; 2: moderately-differentiated, MD; 3: poorly-differentiated, PD), clinical stage (1, 2, 3, or 4), and metastasis status (0, M0; 1, M1). We exclude age from the phenotype data since it is not a strong cancer indicator. We find the cluster nodes either solely with epigenetic data or with the phenotype and epigenetic data together. Because the aim of clustering is to group the samples into different clusters, only one dataset is used. The cluster is validated with Dunn’s index (DI).

### Clustering with kernel *Kmeans*

In this approach, we first find the clusters (nodes) based on epigenetic data and kernel *Kmeans*, and then the node centers are estimated to be the median of the phenotype and genotype within each cluster. We then build the pathway based on nodes heritability. The output for the node centers are given in Table (1).

**Figure 1:**
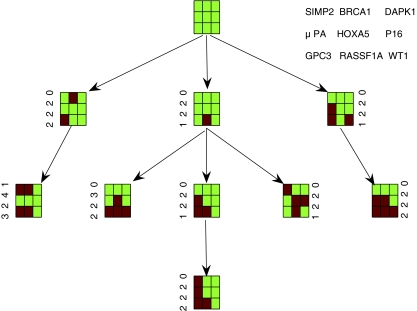
Progression pathway clustered with kernel kmeans.

Based on the node centers, the pathway tree built with the proposed algorithm is given in Figure (1). In Figure (1), the green spots represent the unmethylated loci and the red spots are the hypermethylated loci. The gene name of each locus is shown in the upper right corner of the figure. The numbers beside each node plot are the phenotype centers (medians).

### Fuzzy kernel *Kmeans*

**Figure 2:**
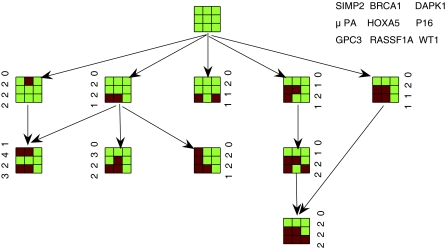
Progression pathway clustered with fuzzy kernel kmeans.

We also found the nodes (clusters) with fuzzy kernel *Kmeans* and genotype data. There is one more cluster (node) in the tumor progression tree and multiple pathways with Fuzzy kernel *Kmeans*. Figure (2) is the corresponding progression pathway.

### Results

The progression Pathways presented both in Figure (1) and Figure (2) validate the notion that tumors with more aggressive phenotypes should have higher level of methylation than that with less aggressive phenotypes. The first phenotype is the Hormone receptor status (ER/PR). The progression becomes more aggressive when more hypermethylation happened among the 9 genes. The other two phenotypes, histology and clinical stage, have the similar trend to progress from lower stage to higher stage in both progression trees. Metastasis happened in the terminal nodes of the pathways.

The hypermethylation in the promoters of GPC3 and RASSF1A happened in several intermediate and terminal nodes of both trees. This is consistent with the observation that a large number of tumors have concurrent hypermethylation of GPC3 and RASSF1A. The progression pathways also indicated that hypermethylation in the promoter of 4 genes: 3OST3B, BRCA1, GPC3, and RASSF1A, leads to the most aggressive tumors whereas methylation in other genes has much less effect. Therefore, genes 3OST3B and BRCA1 should be studied carefully in functional analysis. Another gene HOXA5 may also play an important role in tumor aggression. Fuzzy kernel *Kmeans* seems to perform better in the sense that it finds one more node and multiple pathways.

## 4. Conclusions and Remarks

We proposed an entropy kernel and kernel based algorithm for recapitulating tumor progression pathway and biomarker finding. The proposed fuzzy kernel kmean algorithm seems to perform better than the standard kernel kmean, since the DI for fuzzy kernel *kmeans* (0.52) is greater than that for kernel *kmeans* (0.41). It can also provides multiple pathways. In the constructed progression tree, the progeny nodes have more hypermethylated gene promoters than its parent nodes and the progeny node has more aggressive tumor than its parents. The proposed method can not only discover different tumor progression paths but also find genes (biomarkers) that lead to more aggressive tumors and have biological and clinical significance. Fuzzy kernel Kmean algorithm is a novel idea that is not found in the current literature. The proposed algorithms can be applied to analyze hypomethylation data and build corresponding pathways with little revision. With appropriate kernel functions, the method can be utilized to analyze other sequential and microarray data.

## Figures and Tables

**Table 1:** Node centers of genotype and phenotype data.

Genotype center	phenotype center
111010000	2230
110000110	3241
111110000	2220
010000000	1220
110100100	1220
100000010	2220
110100000	1220
010011100	1220
101100000	1220
